# Dietary diversity and associated factors among children of Orthodox Christian mothers/caregivers during the fasting season in Dejen District, North West Ethiopia

**DOI:** 10.1186/s12986-018-0248-0

**Published:** 2018-02-14

**Authors:** Gemechu Kumera, Endalkachew Tsedal, Mulatu Ayana

**Affiliations:** Department of Public Health, College of Health Sciences and Medicine, Debre Markos University, Debre Markos, Ethiopia

**Keywords:** Dietary diversity, Orthodox Christian, Children, Ethiopia

## Abstract

**Background:**

Proper feeding practices during early childhood is fundamental for optimal child growth and development. However, scientific evidences on the determinants of dietary diversity are scanty. Particularly, the impact of fasting on children`s dietary diversity is not explored in Ethiopia. The aim of this study was to assess dietary diversity and associated factors among children aged 6–23 months, whose mothers/care-givers were Orthodox Christians during the fasting season (Lent), in Dejen District, North West Ethiopia, 2016.

**Methods:**

A community based cross-sectional study was conducted during the fasting season from March to April, 2016. The study sample were children aged 6–23 months, whose mothers/care-givers were Orthodox Christians. A systematic random sampling technique was used to select a sample of 967 children proportionally from all selected kebeles. Data was entered using Epi data and statistical analysis were done using logistic regression. P-value < 0.05 at 95% confidence interval was taken as statistically significant.

**Results:**

Only 13.6% of children surveyed met the minimum requirement for dietary diversity. Unsatisfactory exposure to media [AOR = 5.22] and low household monthly income [AOR = 2.20] were negatively associated with dietary diversity. As compared to economic related reasons, mothers/caregivers who do not feed diet of animal origin to their children due to fear of utensil contamination for family food preparation were 1.5 times [AOR=1.5; 95% CI (1.05 – 2.53)] less likely to feed the recommended dietary diversity.

**Conclusions:**

The findings of this study revealed that the diet of children in the study area lacked diversity. Promoting mass media and socioeconomic empowerment of women have positive contribution to optimal child feeding practice. Sustained nutrition education to mothers regarding proper infant and young child feeding practice in collaboration with the respective religious leaders is highly recommended.

## Background

Proper feeding practice during childhood is fundamental for optimal child growth, healthy life, and development [1]. The World Health Organization(WHO) recommends introducing complementary foods when an infant reaches 6 months of age to meet the nutritional requirements of children [2–7].

Suboptimal child feeding practices contributes to poor physical growth including irreversible outcomes of stunting, poor cognitive development, significantly increased risk of infectious diseases and mortality [8–16]. Globally, out of the 10.9 million under-5 year deaths that occur, malnutrition is, directly or indirectly, responsible for 60.0% of death. Over two-thirds of these deaths are associated with inappropriate feeding practices during the first 2 years of life [6, 9]. More than 3.4 million under-5 year children die each year due to inappropriate feeding practices [17].

Dietary diversity refers to increasing the consumption of a variety of foods across and within the food groups capable of ensuring adequate intake of essential nutrients that can promote health, physical and mental development [18]. Dietary diversity (DD) have been used as a proxy indicator of dietary quality and nutrient adequacy [19–22]. Minimum dietary diversity refers to the proportion of children aged 6–23 months who received at least 4 or more varieties of foods from the seven standard food groups recommended by the WHO on the preceding day without imposing a minimum intake restriction [23].

**Fig. 1:**
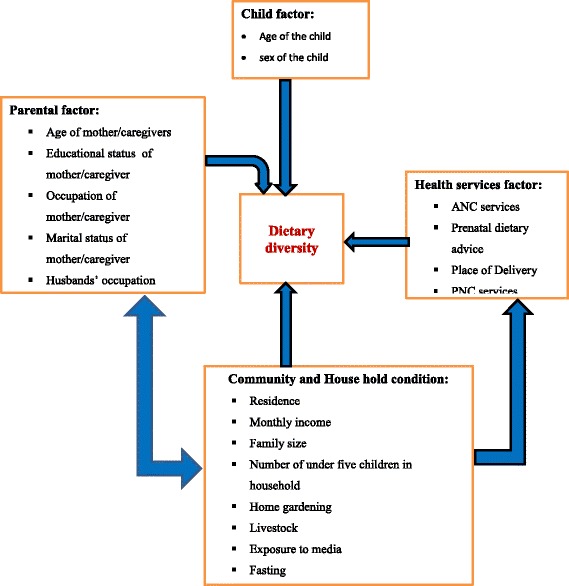
Conceptual framework of factors associated with dietary diversity among children of Orthodox Christian mothers/caregivers during the fasting season in Dejen District, Northwest Ethiopia

Numerous socioeconomic and cultural factors affect feeding practices of children [24, 25]. Societies’ cultural and religious beliefs that has been passed on from generation to generation has a great influence on feeding practices. Beliefs and practices regarding what and how to eat among the community at large and at the household level in specific has a great influence on dietary diversity. Additionally culture, religion and traditional knowledge affect food and nutrition security by shaping communities diet, intra household food distribution patterns, and child feeding practices, which in turn affects dietary diversity score and intake of nutrient rich foods in children [26] (Fig. 1).

Ethiopian Orthodox Christian followers fast a frequency of approximately 250 days a year. In this religion there are seven official fasting periods. All these fasts are obligatory for every Orthodox Christian followers, except children less than 7 years and pregnant mothers. Of these fasts; Lent, at which this study was conducted is observed with greater rigor than any other fast. According to the religion doctrine; during this fasting period, eating animal products like flesh foods, eggs and dairy products are strictly forbidden except children and pregnant women [27].

According to the Ethiopian Demographic and Health Survey (EDHS) 2011, 44% of children are stunted, 9.7% are wasted, and 29% are underweight [28]. Nutritional problems such as stunting in children are strongly associated with the feeding practices [12, 29]. According to Alive and Thrive Ethiopia and EDHS 2011, only 4.8% of the children achieve minimum dietary diversity [30], and only 4.1% meet the criteria for the minimum acceptable diet [28]. A detailed analysis of EDHS 2011 showed that only 10.8% of diets of children aged 6–23 months have met the minimum requirement for dietary diversity [31] whereas, a study in Northwest Ethiopia reported that only 12.6% of diets of children met the minimum requirement for dietary diversity [32]. Other studies conducted in Democratic Republic of Congo, Burkina Faso, Mali, and India also reported low prevalence of dietary diversity [33, 34].

This evidence calls strongly for the need to improve feeding practices of children. However empirical evidence on the dietary diversity and associated factors are scanty. Particularly, the impact of fasting on children’s dietary diversity is not explored in Ethiopia. Updated knowledge on dietary diversity will assist the national nutrition program to better monitor the changes in the feeding practices [35]. Information on the dietary diversity and associated factors are also needed for prioritizing, designing and initiating further intervention programs aimed at improving dietary diversity and thereby contribute in reducing under nutrition in children. Thus, the objectives of the current study are to assess dietary diversity and associated factors among children aged 6–23 months in Dejen district, Northwest Ethiopia. The current study also assessed the potential impact of mothers/care-givers fasting on children’s dietary diversity which was not explored by earlier studies in Ethiopia [31, 32].

## Methods

### Study design, area and period

A community based cross-sectional study was conducted during Lent (fasting season) from March to April, 2016. The study was conducted in Dejen district in North West Ethiopia. According to the 2015/2016 Ethiopian census, the total population of the district was estimated to be 119, 454 from which 5184 were children aged between 6 and 23 months whose mothers were Orthodox Christians (96% of the total population) [36, 37]. The district comprises 2 urban and 21 rural kebeles (a kebele is the smallest administrative unit in Ethiopia, comprising approximately 1000 households). Urban population accounted for 9.8% (11,758) of the total district population. The study sample was children aged 6–23 months whose mothers/care-givers were Orthodox Christians living in the Dejen district.

### Sample size and sampling technique

Sample size for estimating the proportion of minimum dietary diversity was computed using single population proportion sample size calculation formula with the inputs of 95% confidence level, 2% of margin of error, non-response rate of 10% and expected proportion of minimum dietary diversity 12.6% (previous research conducted at Dangila town, Northwest Ethiopia) [32]. All 23 kebeles in the district were stratified into two - 2 urban and 21 rural. Simple random sampling technique was used to select one urban kebele and ten rural kebeles from each strata. Sampling frame was developed for each kebeles by having a house to house enumeration. A list of children aged 6–23 months residing in the selected kebeles was obtained from health extension workers. The total sample size was allocated to the two strata proportionally to their population size. The sample size for each stratum was allocated to the kebeles proportional to their population size. Ultimately 967 children aged 6–23 months were selected from the list of children using systematic random sampling technique.

### Data collection procedures

Data on potential determinants of dietary diversity were collected from the mothers/caregivers of the selected children using structured questionnaire. Data on sociodemographic factors, community and house hold conditions, health services factors and dietary intake were collected. The parts of the questionnaire on dietary diversity (DD) was adopted from WHO standardized questionnaire for Infant and young child feeding (IYCF). Other parts of the data collection tool were adopted from EDHS 2011 [28] and developed by the principal investigators. The questionnaire was administered in local language(Amharic). The English version of the questionnaire was translated to local language and back to English by an expert to insure its consistency, and locally available foods were also considered. The translated Amharic version (local language) was pretested prior to the actual survey and modifications were made accordingly. The content validity of the questionnaire was assessed against the conceptual framework of the study. Principal investigators reviewed all of the questionnaire items for readability, clarity, accuracy of the knowledge measured and comprehensiveness and come to some level of agreement as to which items should be included in the final questionnaire. Reliability of the tool was checked using test-retest method. Questions with less than 0.7 kappa or Pearson coefficient values were removed or revised. Two days intensive training was given for data collectors and supervisor by the principal investigators to have consensus and the same understanding of what is intended to be measured by each question in the questionnaire, and how to maintain ethical issue. The data collection process was followed daily by the supervisor and principal investigators to assure quality of the data. Data were collected using interviewer- administered questionnaire. Interviews were conducted when the mother/caregiver was expected to be home, in the mother’s/caregiver’s home.

### Operational definition

#### Minimum dietary diversity

The WHO recommended infant feeding guidelines were used for measuring dietary diversity. The dietary diversity was assessed using 24-h recall method. This was based on the mother’s/caregiver’s recall of foods given to her child in the past 24 h prior to the interview date. Mothers/caregivers were asked to recall foods given to her child from predefined seven food categories in the previous day of the survey. A dietary diversity score was computed by summing consumption of seven types of food groups: grains, roots and tubers; vitamin-A-rich fruits and vegetables; other fruits and vegetables; legumes and nuts; dairy products (milk, yogurt); flesh foods (meat, fish, poultry and liver/organ meats) and eggs. From the dietary diversity score, the minimum dietary diversity indicator was constructed using the WHO recommended cut-off point with a value of “1” if the child had consumed four or more groups of foods and “0” if less. Consumption of any amount and quality of food from each food group was sufficient to ‘count’, i.e., there was no minimum quantity, except if an item was only used as a condiment. Accordingly, minimum dietary diversity was defined as the proportion of children who received at least 4 or more varieties of foods from the seven food groups in a 24-h time period [23].

#### Satisfactory exposure to media

Mothers/caregivers of the children at least once a week read a newspaper or magazine or listen to radio, or watched television. Less exposure than once a week was defined as unsatisfactory exposure to media.

#### Nutrition education

Nutrition education was assessed using the items adapted from the national nutrition survey conducted by the Ethiopian Health and Nutrition Institute in 2009. Nutrition education was constructed by combining three items that asked the mother/caregiver whether she received each of the following advices: [1] advised to eat a balanced diet, [2] advised to eat more and [3] advised to eat different fruits and vegetables. A value of 1 was assigned if the pregnant woman answered yes to each item and 0 was assigned otherwise. The scores of nutrition education were recorded to develop dichotomous variable that divided the respondent who received any prenatal dietary information (nutrition education) and those who did not.

### Data processing and analysis

Data analysis was performed using SPSS version 20. Descriptive analysis was done using mean, frequency and percentage. The association between the dependent and independent variables reported in univariate analysis was further examined by using multiple logistic regression after controlling for potential confounders in the model. The stepwise backward elimination procedure was used in the multiple logistic regression. The collinearity effect was tested using the Variance Inflation Factor (VIF) for all independent variables. Model fitness was assessed using the Hosmer-Lemeshow statistic test. *P*-value < 0.05 at 95% CI was considered statistically significant.

### Ethical consideration

The study was approved by the ethical review boards of College of medicine and Health Science, Debre Markos University prior to data collection. Support letters were obtained from Dejen administrative council and health office. Verbal consent was obtained from parents before enrolment in the study after the nature of the study was fully explained to the parents. Study participants (mothers/care-givers) were told that they had full right to participate or not, and they were also informed that all the data obtained from them would be kept confidential using codes instead of any personal identifiers. Nutrition education was given to all study participants.

## Results

### Socio-demographic characteristics of the study participants

Table 1 summarizes socio-demographic characteristics of the study participants. Of 967 children sampled, 955 were participated in the study. Accordingly, the response rate was 98.8%. The mean age (+/−standard deviation) of the respondents (mothers/caregivers) was 29 years (+/− 7.9 years). The majority of the respondents, 889(93.1%) were rural dwellers. Nearly half, 438 (45.9%) of the respondents had no formal education, and more than four-fifth, 818(85.7%) were farmers. Of the children included in the study, 466(48.8%) were males and 489(51.2%) were females. The average household size was 6.3(+/− 0.7). The mean household monthly income was 456.37 Ethiopian Birr (ETB).

**Table 1:** Parental characteristics of children aged 06–23 months, in Dejen District, Northwest Ethiopia, 2016

Characteristics		Frequency (n)	Percent (%)
Age of mothers	≤ 29 > 29	575 380	60.2 39.8
Mothers’ educational status	Unable to read and write Primary High school Certificate and above	438 370 120 27	45.9 38.7 12.4 2.8
Mothers’ occupation	Farmer Merchant Government employee Others^a^	818 82 27 28	85.7 8.6 2.8 2.9
Husbands’ occupation	Farmer Merchant Government employee Others^b^	787 71 26 11	87.9 7.9 2.9 1.2
Age of the child	6–12 months 12–23 months	357 598	37.4 62.6
Residence	Urban Rural	66 889	6.9 93.1
Sex of last child	Male Female	466 489	48.8 51.2
Number of under five children	One Two	949 6	99.4 0.6
Family size	≤ 4 > 4	529 426	55.4 44.6
Marital status	Married Others^c^	895 60	93.7 6.3
Monthly income	< 456 Ethiopian Birr ≥ 456 Ethiopian Birr	568 387	59.5 40.5

^a^housewife/student, ^b^daily laborer ^c^single/divorced/widowed

### Maternal health and feeding practices

Table 2 summarizes the maternal health and feeding practices. Majority of the respondents (mothers/caregivers), 943(98.7%) received antenatal care (ANC) at least once during their last pregnancy, but only 478(50%) had four or more ANC visits (which is recommended). More than half of the respondents, 594(62.2%) were eating at least one additional food during their last pregnancy period. More than three quarters of the respondents, 748(78.2%) gave birth at health institution during their respective last delivery and significant number of respondents, 109(11.4%) reported home delivery. Majority, 891(93.3%) of the mothers reported that they got postnatal care service during the 7 days following their last delivery.

**Table 2:** Maternal health and feeding practices in Dejen district, Northwest Ethiopia, 2016

Characteristics		Frequency (n)	Percent (%)
Number of ANC visit	One Two Three > = 4	47 212 219 478	4.9 22.2 22.9 50.0
Nutrition education during pregnancy	Yes No	880 75	92.2 7.8
Feeding habit during pregnancy	Increased Similar Reduced	594 261 100	62.2 27.3 10.5
Place of delivery	Hospital/Health center Health post Home	748 98 109	78.2 10.4 11.4
Nutrition education after delivery	Yes No	877 78	91.8 8.2
Feeding habit after delivery	Increased Similar Reduced	679 246 30	71.1 25.8 3.1
Exposure to media	Satisfactory Unsatisfactory	240 715	25.1 74.9
Home gardening	Yes No	124 831	13.0 87.0
Reason not to feed animal products	Due to economic issue Due to fasting	410 473	46.4 53.6

All, 955(100%) of the respondents were fasting during Lent (fasting season). A significant number, 307(32.1%) of households had at least one cow that gives milk during data collection period but only, 48(15.6%) fed milk and/ or milk products to their respective children in the last 24 h prior to the data collection period. The main reason not to feed milk and/or milk products were due to fear of utensil contamination during family food preparation 173(66.8%), the milk was not churned (butter or fat part of milk was not separated) 80(30.9%) and due to poor attention 6 (2.3%). One hundred seventy seven (18.5%) of households had also at least one hen that lays egg during data collection period. Of these households, only 10 (5.6%) fed egg to their children in the last 24 h prior to the data collection period. Fear of utensil contamination during family food preparation (92.2%) was the main reason not fed egg to their children.

All, (100%) of the mothers were feeding breast to their respective children during data collection period. All children, (100%) were started complementary feeding during data collection period and more than three quarters, 727 (76%) started complementary feeding by the age of 6 months. During the introduction of additional foods, more than half (61.2%) of the mothers had given porridge to their children.

### Dietary intakes of the study participants

Table 3 presents the types of food fed to the children. The diet of the study participants (children) was mainly based on grains (98.1%), and legumes and nuts (87.9%). The mean Dietary Diversity Score (DDS) was 3.13 (± 0.87) ranging between 1 and 6. The majority of study participants, (86.4%) had low DDS (< 4 food groups). Small proportion (7.5%) of study participants reported that they consumed diet of animal origin the day before the survey. Among animal products flesh meat was totally not consumed by the study participants, whereas egg, milk and milk products were consumed by (2%) and (7.5%), respectively.

**Table 3:** Types of food groups fed to the children in Dejen district, Northwest Ethiopia, 2016

Characteristics		Frequency (n)	Percent (%)
Grains, roots and tubers	Yes	937	98.1
	No	18	1.9
Legumes and nuts	Yes	839	87.9
	No	116	12.1
Dairy products	Yes No	72 883	7.5 92.5
Flesh food	Yes No	0 955	0 100
Eggs	Yes No	936 19	98.0 2.0
Vitamin A rich fruits and vegetables	Yes No	167 788	17.5 82.5
Other fruits and vegetables	Yes No	749 206	78.3 21.7

### Factors associated with dietary diversity

Table 4 summarizes factors associated with dietary diversity. Independent variables that showed association on the bivariate logistic regression model include: residence, monthly income, educational status, maternal feeding habit during pregnancy, maternal feeding habit after delivery, media exposure and reason not to feed diet of animal origin. The multivariable logistic regression analysis revealed that monthly income, media exposure and reason not to feed diet of animal origin were variables which significantly associated to dietary diversity.

**Table 4:** Factors associated with dietary diversity among children aged 06–23 months, in Dejen District, North West Ethiopia, 2016

Predictors	Dietary diversity score		COR (95%CI)	AOR (95% CI)	P- values
	< 4	≥ 4			
Residence					
Rural	783	106	4.22(2.46, 7.25)	1.67(0.69, 4.01)	0.25
Urban	42	24	1.0	1.0	
Educational status					
No formal education	395	43	1.0	1.0	
Primary education	325	45	1.27(0.82, 1.98)	0.76(0.41, 1.42)	0.4
High school and above	105	42	2.28(2.28, 5.92)	1.20(0.57, 2.49)	0.63
Feeding habit during pregnancy					
Increased	242	19	1.0	1.0	
Same or reduced	503	92	2.33 (1.39, 3.91)	1.4(0.7, 2.8)	0.34
Feeding habit after delivery					
Increased	225	21	1.0		
Same or reduced	573	107	2.0(1.22, 3.27)	1.99(0.86, 4.6)	0.11
Media Exposure					
Unsatisfactory	668	47	7.51(5.05, 11.8)	5.22(3.29, 8.26)	0.001^**^
Satisfactory	157	83	1.0	1.0	
Monthly Income					
< 456 Ethiopian Birr	521	47	3.03(2.06, 4.45)	2.20(1.39, 3.49)	0.001^**^
≥ 456 Ethiopian Birr	304	83	1.0	1.0	
Reason not to feed diet of animal origin					
Due to economic issue	359	51	1.0	1.0	
Due to fasting status	435	38	1.63(2.01, 3.84)	1.50(1.39. 3.49)	0.02^*^

^*^P-value is significant at < 0.05. ^**^P-value is significant at < 0.01

Mothers/caregivers who had been exposed to media had a higher odd of feeding the recommended dietary diversity to their children [AOR = 5.22; 95% CI (3.29–8.26), *P* = 0.001] than those mothers/caregivers who had not been exposed to media.

Monthly income was found to be significantly associated with dietary diversity. Mothers/caregivers with low monthly income (< 456.4 ETB) were two times more likely to feed below the recommended dietary diversity to their children [AOR = 2.2; 95% CI (1.39–3.49), P = 0.001) as compared to those with high monthly income.

The current study indicated that as compared to economic related reasons, mothers/caregivers who do not feed diet of animal origin to their children due to fear of utensil contamination for family food preparation were 1.5 times [AOR = 1.5; 95% CI (1.05–2.53)] less likely to feed the recommended dietary diversity.

## Discussion

This study assessed dietary diversity and associated factors among children aged 6–23 months, whose mothers/care-givers were Orthodox Christians during the fasting season (Lent). The study reveals that the diet of children in the study area was mainly composed of grains, legumes and nuts. The majority of the children only consumed foods from less than four different food groups the day before the survey which is below the WHO recommendation for children’s dietary diversity [23]. Diet of animal origin like meat, egg and dairy products were a rare component in the children’s diet.

In the current study, only 13.6% of the children surveyed met the minimum requirement for dietary diversity. This finding was comparable with studies conducted in Ethiopia [31, 32], Democratic Republic of Congo (12%), Burkina Faso (14%), Mali (16%) [33], and India (15.2%) [34]. However, it is lower than other reports from India (33%) [38], Nepal (30.4%) [39], Bangladesh (42%) [40], Sri Lanka (71%) [41] and Nepal (72%) [42]. This variation might be due to the lack of awareness about child feeding practices, traditional variation in feeding habits and cooking a few varieties of food for the family, low purchasing power of food and seasonal difference in data collection. Further, the role of religious traditions in the Ethiopian diet is very relevant. The Ethiopian Orthodox Church prescribes certain periods of fasting, which include all Wednesdays and Fridays as well as several long periods spread over the year, approximately 250 days (68.4%) of a year. According to the doctrine, during fasting days or periods only one meal per day is permitted, and the consumption of diet of animal origin is strictly forbidden [27, 43]. For this reason, mother/care givers are not willing to prepare non fasting foods for their children during the fasting season because they fear that it will contaminate utensils used for cooking family foods. Moreover, during fasting season meat and chicken are not available in the market and slaughtering any animal is not allowed.

During the last 2 years the government and different Nongovernmental organizations(NGOs) such as Alive & Thrive, USAID and UNICEF had given a great emphasis on infant and young child feeding practices [43]. During this time, community mobilization about proper infant and young child feeding practice were highly taking place in the area. If it was not fasting season, DDS would be more than reported in the current study. Moreover, this district is one of the districts which has surplus production and a diversified food item produced; as a result, they had a chance to achieve a minimum dietary diversity even excluding animal products.

The study finding indicated that as compared to economic related reasons, mothers/caregivers who did not feed diet of animal origin to their child due to fear of utensil contamination because of the fasting season were less likely to feed the recommended dietary diversity. Previous study conducted in Tigray region, also supported the finding [44]. This might be due to the reason that both areas shared similar religious characteristics and both of them studied in the fasting season though the one, which was done in Tigray was not during Lent. Additionally, in both areas misunderstanding about feeding animal products during the fasting season might affect dietary diversity of children.

The current study finding witnessed significant positive association between mothers’ media exposure and dietary diversity of children. Previous studies conducted in Ethiopia [31, 32], India [34] and Sri Lanka [41] have also documented similar findings. This might be due to the reason that media exposure might have contributed to optimal child feeding practices through enhancing good nutritional awareness and practice of mothers/caregivers. Mothers/caregivers who read a newspaper or magazine or listen to the radio, or watched television are more likely to get education on IYCF practice provided through mass media. Mass media influence the mother’s knowledge and behavior in matters related to their children feeding practices and health [45–47].

Another important determinant of the dietary diversity of children in the study area is household monthly income. Those with low monthly income were less likely to feed the recommended dietary diversity to their children as compared to their high monthly income counterparts. This finding is consistent with study conducted in Cambodia [48]. This may indicate that family income has a direct association with household food security, since food consumption is believed to be heavily influenced by income level. Moreover, socioeconomic factors operate indirectly to influence children’s dietary intake by determining the quality of a child’s diet and also affects the ability to access food.

Major strengths of this study were the random selection of the study participants and the collection of data from the community. As the study was community based and the study participants were selected randomly representativeness can be assured. The major limitation of the current study was the cross sectional nature of its design as we can’t establish causal relationships between the independent variables and dietary diversity of the children. Secondly, assessment of children’s dietary intake depends on the 24-h recall method, which may not accurately reflect their past feeding experience. Third, quality and the amount of food given for children were not taken into account.

## Recommendations

National level study with strong study design should be conducted in Ethiopia. The promotion of dietary diversification strategies to improve children’s food consumption is needed in the study area. Promoting the socioeconomic empowerment of women and higher agricultural productivity have positive contribution. Sustained nutrition education to mothers regarding proper child feeding practices in collaboration with the respective religious leaders is highly recommended. We also suggest programmers to increase mass media coverage and accessibility.

## Conclusion

The findings of this study reveal that children diet lacked diversity according to WHO recommendations. Small proportion (13.6%) of children met the recommended minimum dietary diversity. The diet of the children was mainly based on grains (98.1%) and legumes (87.9%). Mother’s/caregiver’s fasting practice affected the dietary diversity score of the children. Low household economic status, unsatisfactory mothers’ exposure to media and fear of utensil contamination for family food preparation are key predisposing factors to low dietary diversity.

## Abbreviations

ANC: Ante Natal Care
DD: Dietary Diversity
DDS: Dietary Diversity Score
EDHS: Ethiopian Demographic and Health Survey
ETB: Ethiopian Birr
IYCF: Infant and young child feeding
NGO: Non-Governmental organization
OR: Odds Ratio
SD: Standard Deviation
USAID: United States Agency for International Development
WHO: World Health Organization
